# Interstitial cells of Cajal link excitatory and nitrergic inhibitory neurotransmission with slow-wave activity in the intestine

**DOI:** 10.1186/2050-6511-14-S1-O30

**Published:** 2013-08-29

**Authors:** Sabine Klein, Barbara Seidler, Anna Kettenberger, Andrei Sibaev, Michael Rohn, Robert Feil, Hans-Dieter Allescher, Jean-Marie Vanderwinden, Franz Hofmann, Michael Schemann, Roland Rad, Martin A Storr, Roland M Schmid, Günter Schneider, Dieter Saur

**Affiliations:** 1 Department of Internal Medicine 2, Ludwig-Maximilian Universität München, Germany; 2 Department of Internal Medicine 2, Ludwig-Maximilian Universität München, Germany; 3 Lehrstuhl für Humanbiologie, Ludwig-Maximilian Universität München, Germany; 4 University of Tübingen, Interfaculty Institute of Biochemistry, Tübingen, Germany; 5 Zentrum für Innere Medizin, Klinikum Garmisch-Partenkirchen, Garmisch-Partenkirchen, Germany; 6 Laboratory of Neurophysiology, Faculty of Medicine, Université Libre de Bruxelles, Brussels, Belgium; 7 Forschergruppe 923, Institut für Pharmakologie und Toxikologie; Klinikum rechts der Isar, Technische Universität München, Ismaningerstr. 22, 81675 München, Germany; 8 Welcome Trust Sanger Institute, Genome Campus, Hinxton-Cambridge CB10 1SA, UK

## Introduction

Interstitial cell of Cajal (ICC) form a cellular network that is embedded in the musculature of the gastrointestinal tract. Previous studies provided clear evidence that ICC generate a rhythmic pacemaker current which manifests itself as slow-waves in membrane potential of smooth muscle cells resulting in rhythmic bowel contractions. However, a role of ICC in the transmission of excitatory and inhibitory signals from enteric neurons to smooth muscle cells is highly controversial and remains an outstanding question. This is mainly due to the general lack of models and systems to study ICC function in adult animals at the level of genetics. The aim of this study was to investigate the role of ICC for excitatory and inhibitory nitrergic neurotransmission in the gut.

## Materials and methods

Using genetic engineering in mice, we generated an inducible *c-Kit^CreERT2^* knock-in allele at the endogenous *c-Kit* locus which enables for the first time genetic manipulation and depletion of ICC in vivo at defined time points during development and in adults by tamoxifen administration. With the help of this novel model, we depleted ICC by using a conditional *LSL-R26^DTA/^*^+^ mouse line, which carry a latent diphtheria toxin A (DTA) expression cassette. Furthermore, we deleted *cGMP-dependent protein kinase I* (*Prkg1*), the central mediator of the non-adrenergic, non-cholinergic neurotransmission in ICC using floxed *Prkg1* animals.

## Results

Tamoxifen induced disruption of the ICC network in healthy adult animals resulted in severely disturbed GI motility with significantly increased GI transit time. Organ bath experiments and intracellular recordings revealed dysrhythmic spontaneous phasic myogenic contractions and lack of slow-wave type electrical activity in circular small intestinal smooth muscle cells. After electrical field stimulation neuronal induced colonic smooth muscle contractions were significantly impaired in tamoxifen treated animals. Deletion of *Prkg1* in ~40% of all ICC resulted in a severely disturbed GI motility and abolished specifically the NO-dependent slow inhibitory junction potential in colonic circular smooth muscle cells.

## Conclusion

ICC integrate excitatory and inhibitory nitrergic neurotransmission with slow-wave activity to orchestrate peristaltic motor activity of the gut. Impairment of ICC function causes severe gastrointestinal motor disorders. The results of our study show at the genetic level that these disorders are not only due to loss of slow-wave activity but also due to disturbed neurotransmission.

